# Solvent Dependency of Sorghum Bran Phytochemicals Acting as Potential Antioxidants and Antibacterial Agents

**DOI:** 10.17113/ftb.59.01.21.6878

**Published:** 2021-03

**Authors:** Varee Tyagi, Chakkaravarthi Saravanan, Yixiang Wang, Bhaswati Bhattacharya

**Affiliations:** 1 Department of Basic and Applies Sciences, National Institute of Food Technology Entrepreneurship and Management, Sector - 56, HSIIDC Industrial Estate, Kundli, Sonipat, 131028 Haryana, India; 2 Department of Food Science and Agricultural Chemistry, McGill University, 21111 Lakeshore, Ste Anne de Bellevue, H9X 3V9 Quebec, Canada

**Keywords:** sorghum bran, ionic liquid, phytochemicals, antioxidant activity, antibacterial activity

## Abstract

**Research background:**

Sorghum bran, although considered as an agricultural waste, is an abundant source of various bioactive compounds. These bioactive compounds require specific extraction with particular solvents and therefore ionic liquid and three different conventional solvents, *viz.* anhydrous methanol, acidified methanol and water were used in this work.

**Experimental approach:**

To evaluate the phytochemicals in the different sorghum bran extracts, total phenol content, flavonoids, condensed tannins and anthocyanins were determined as per standard protocols. Liquid chromatography with tandem mass spectrometryanalysis of extracts was also performed for their phenolic profiling. The antioxidant activity of the extracts was estimated *via* three assays: 2,2-diphenyl-1-picrylhydrazyl (DPPH) free radical scavenging assay, 2,2-azino-bis(3-ethylbenzothiazoline-6-sulfonic acid) (ABTS) radical cation decolourization assay and Cu^2+^ reducing antioxidant capacity (CUPRAC) method. The antibacterial activity against two most opportunistic foodborne pathogens: *Escherichia coli* and *Staphylococcus aureus* was measured by agar well diffusion assay and minimum inhibitory concentration (MIC) was determined by serial dilution method.

**Results and conclusions:**

Ionic liquid extract of sorghum bran gave the highest yield ((14.9±0.7) %), which indicated that various possible interactions like Van der Waals forces, H-bonding, hydrophobic and cation-π bonding can occur when ionic liquid is used as an extractant compared to other conventional solvents, although total phenol mass fraction expressed as gallic acid equivalents on dry mass basis was only (7.4±0.7) mg/g. The hydrophobicity of the ionic liquid also helped in efficient extraction of condensed tannins ((63.2±2.1) mg/g expressed on dry mass basis), which resulted in significant antioxidant activity of the ionic liquid extract ((85.2±1.2) µmol/g in DPPH assay, (100.8±0.9) µmol/g in ABTS assay and (63.2±1.9) µmol/g in CUPRAC). An interesting revelation reported in this work is the inability of DPPH assay to evaluate the antioxidant activity in acidic environment. The anhydrous methanolic extract of sorghum bran displayed pH sensitivity, making the extract beneficial for certain applications. Qualitative analysis of extracts revealed greater number of phenolic compounds to be present in methanol and distilled water extracts. Moreover, various derivatives of apigenin and luteolin were also observed in all four extracts. In addition, the acidified methanol extract of the sorghum bran exhibited antimicrobial property at a concentration of 12 mg/mL. A larger inhibition zone was observed against *Escherichia coli* than *Staphylococcus aureus,* while the MIC against these two bacteria was 2.2 and 1.1 mg/mL, respectively.

**Novelty and scientific contribution:**

This paper presents the first information on the application of ionic liquids as extracting phase for sorghum bran polyphenols and the quantification of such extracts. As evident from the study, each solvent has its own role in the extraction of bioactive compounds. This work also proves that sorghum bran imparts antibacterial activity against foodborne pathogens.

## INTRODUCTION

Crop plants add value to the earth's diversity and are fundamental to all life. They include high content of non-nutritive and bioactive compounds such as flavonoids, phenolics, anthocyanins, phenolic acids, and nutritive compounds such as sugars, essential oils, carotenoids, vitamins and minerals (*1*, *2*). Sorghum contains a broad spectrum of polyphenols, classified as phenolic acids, tannins and flavonoids, where the flavonoids are further categorised as anthocyanins. In some sorghum varieties, phenolic acid content was observed to be in the range of 135.5-479.4 μg/g, mainly comprising protocatechuic and ferulic acids (*3*). Flavonoid content in sorghum largely comprises 3-deoxyanthocyanidins (79%), of which luteolinidin and apigenidin are the main non-methoxylated forms of 3-deoxyanthocyanidins (*3*). Tannins are secondary metabolites that provide protection against pathogens and predators. Usually, the tannins in sorghum have high molecular mass, degree of polymerisation of more than 10 and their content varies between 0.2 and 48 mg/g. Sorghum tannins are classified as type I (not significant), type II (extractable in acidified methanol) and type III (extractable in methanol and acidified methanol), and are generally condensed in nature, typically constituting oligomers or polymers of catechin (*3*, *4*). These phenolic compounds are mainly concentrated in the sorghum bran (*5*, *6*), which is a by-product of sorghum grain dry milling, and generally considered as an agricultural waste. Sorghum bran had three times higher total phenol content (*7*) and 3-4 times higher anthocyanin content, varying between 3.6-10.1 mg/g (*5*), than the kernel.

To characterize the polyphenols, it is important that their extraction method should be efficient and that it largely depends on the nature of the selected solvent. Polyphenols/anthocyanins are extracted from sorghum bran mainly by aqueous acetone and acidified methanol (*4*, *5*), where acidified solvent extracts have been found to have greater phenolic and flavonoid content with better antioxidant activity. Other than the above conventional solvents, recently various alternative methods that are environmentally friendly have been explored to fasten the extraction. Accelerated solvent extraction (*8*) and ultrasound-assisted extraction (*9*, *10*) of sorghum bran polyphenols have been shown to have higher yield of polyphenolics than conventional methods. Similarly, subcritical extraction method also showed higher yield of polyphenolics, radical scavenging activity and antiproliferative activity against HepG2 cell line than hot water extraction method (*11*). Ultrasound-microwave-assisted (UMA) extraction utilizing different solvents has also been studied (*12*), which gave better yields of sorghum husk extracts.

In line with the above, ionic liquids (ILs), a new class of solvents, have been chosen to be the green alternatives to the conventional extraction solvents. Although there are contrary views on the green aspect of the ionic liquids, these organic salts in liquid state are preferred over conventional solvents due to their non-flammability and low vapour pressure in the role of an extraction solvent. In a recent study, eight different imidazolium-based ionic liquids were used as the solvent for extracting flavonoids from grape skin and compared with conventional extraction solvents (*13*). That study revealed that the structure of cation and anion in the ionic liquid as well as their concentration played a major role in obtaining the final yield. Although organic solvents (*viz.* methanol, ethanol, acetone, *etc.*) have proven their efficiency in the extraction of polyphenols from sorghum, there are no reports of ionic liquids being explored for the extraction of sorghum bran. Furthermore, in one of the previous studies (*14*), it has been observed that 1-ethyl-3-methylimidazolium bromide provides higher yield of polyphenols. Therefore, with an aim of obtaining higher phenolic content, in the current work one of the simplest representatives of cation-based class of ionic liquids, 1-butyl-3-methylimidazolium chloride ([BMIM]Cl) was chosen.

Another focus of this study is the investigation of antimicrobial activity of sorghum bran extracts, which has not been explored until today. However, there are reports available on the antimicrobial activity of sorghum grain extract. Antibacterial activity of sorghum saponin extract has been investigated against *Staphylococcus aureus*, *Escherichia coli* and *Candida albicans*, where its antibacterial activity was proven only against *S. aureus* (*15*). Methanolic extracts of sorghum polyphenols showed no activity against *B. subtilis*, but had inhibitory activity against *Salmonella* spp., *Pseudomonas* spp. and *E. coli* (*16*). Antimicrobial activity of sorghum grains against fungi such as *Alternaria alternata* and *Aspergillus flavus,* along with Gram-negative (*Enterobactor* spp., *Pseudomonas* spp., *E. coli* and *Salmonella* spp.) and Gram-positive (*Bacillus* spp*., S. aureus* and *S. epidermidis*) bacteria has also been proven (*17*). Another study demonstrated antibacterial activity of various solvent extracts of sorghum distillery residues against *E. coli*, *S. aureus*, *Salmonella* spp. and *B. cereus* (*18*).

Thus, the present work focuses on the determination of the yield, study the different phytochemical properties like total phenolic content, total flavonoid content, anthocyanin content, condensed tannins and antioxidant activity of the sorghum bran extracted by 1-butyl-3-methylimidazolium chloride ([BMIM]Cl) and its comparison with conventional solvents like acidified methanol, anhydrous methanol and water. Qualitative analysis (LC/MS/MS) of all the extracts has also been undertaken in this study to further shed light onto the phenolic composition of the extracts. This work also aims to investigate the antibacterial activity of sorghum bran against the most common foodborne microorganisms (*E. coli* and *S. aureus*), usually present in raw/undercooked meat and seafood. Moreover, this investigation attempts to draw a correlation between the different parameters for a smooth understanding of the effect of the solvents in the process of extraction of the different phytochemicals. So far, there are no reports available on the use of anhydrous solvent as a solvent for the extraction of bioactive components from the sorghum bran and hence anhydrous methanol as one of the extraction solvents was included in the present study. Another aspect was also taken into consideration here regarding the role and interaction of various bioactive components present in the crude extract that may give superior functional properties than individual purified components (*19*).

## MATERIALS AND METHODS

### Materials

*Sorghum bicolor* (L.) bran was procured from a cereal grain miller in Gurgaon, Haryana, India.2,2’-Azinobis(3-ethylbenzothiazoline-6-sulfonic acid) (ABTS) diammonium salt, Trolox, Folin-Ciocalteu reagent, 1-butyl-3-methylimidazolium chloride ([BMIM]Cl), gallic acid, catechin, butanol, neocuproine, luteolin (85%), apigenin (95%) and quercetin were obtained from Sigma-Aldrich, Merck, Mumbai, Maharashtra, India. The 2,2-diphenyl-1-picrylhydrazyl (DPPH) was acquired from Acros Organics, Mumbai, Maharashtra, India. Other chemicals such as methanol, ammonium acetate, iron(III) ammonium sulphate, copper chloride, hydrochloric acid, aluminium chloride and sodium carbonate were of analytical grade and acquired from SRL Pvt. Ltd., Gurugram, Haryana and Hi-Media, Mumbai, Maharashtra, India. Nutrient agar and eosin methylene blue were procured from Sigma-Aldrich, Merck. Pure cultures of *Escherichia coli* (ATCC5922) and *Staphylococcus aureus* (NCDC109) were obtained from the Microbiology laboratory, Department of Basic and Applied Sciences, National Institute of Food Technology Entrepreneurship and Management, Sonipat, Haryana, India. Distilled water used in the extraction was prepared in a laboratory using 3361 distillation unit (Borosil, Gurugram, Haryana, India).

### Sorghum bran extraction

Sorghum bran extracts were prepared according to Awika *et al.* (*20*). Four solvent systems were used for the extraction: water, anhydrous methanol, acidified methanol and 1*%* [BMIM]Cl in methanol. Sorghum bran (2.5 g) was added to the different solvents (20 mL) in centrifuge tubes and shaken at low speed for 2 h in an incubator shaker (Labline, Mumbai, Maharashtra, India) at 25 °C. The samples were then stored overnight in the dark at -20 °C. Next, the samples were centrifuged (3-18K centrifuge; Sigma-Aldrich, Merck, Osterode, Germany) at 7000×*g* for 10 min and supernatants of respective solvents were collected. The centrifugation was performed twice with additional washing of samples with their respective solvents (10 mL). The supernatants were collected and stored at -20 °C (TSX40086A ultra-low freezer; Thermo Fisher Scientific, Pittsburgh, PA, USA) for further analysis. Before analysis, the solvents were removed by heating at 40-60 °C and dried matter was weighed.

### Effect of pH variation on sorghum bran extracts

In order to assess the stability of the sorghum bran extracts at different pH values, extract samples were added to the solutions in the pH ranges 1-10. The solutions were prepared using 0.1 M NaOH and 0.1 M HCl. The colour change of each sample was observed with the naked eye.

### LC-MS/MS analysis

Sorghum bran extracts were qualitatively analysed by triple quadrupole LC/MS/MS system (QSight 220; PerkinElmer, Boston, MA, USA). The optimal MS conditions were (*21*–*24*): scan range *m*/*z*=100–900, negative ionization mode, ion source electrospray ionization (ESI), drying gas 80 Pa, nebulizer gas 120 Pa, hot surface induced desolvation (HSID) temperature 250 °C. The sample concentration and infusion flow rates were 200·10^-9^ mg/L and 50 µL/min, respectively.

### Total phenolic content

Total phenolic content (TPC) of the sorghum bran extracts was quantified with the Folin-Ciocalteu method (*25*). Briefly, distilled water (2.8 mL) and 2% sodium carbonate (2 mL) were added to individual sample extracts (0.1 mL) along with Folin-Ciocalteu reagent (0.1 mL) before incubating for 30 min in the dark. The absorbance of the samples and standards was measured at 750 nm (UV-2600; Shimadzu, Kyoto, Japan). Standard curve for analysing TPC of the samples was prepared by using a stock solution of 1 mg/mL gallic acid. The samples were expressed in mg gallic acid equivalents (GAE) per g sample on dry mass basis.

### Total flavonoid content

Total flavonoid content (TFC) of the bran extracts was determined using aluminium chloride colourimetric method (*25*). To summarize, distilled water (2.8 mL), 95% ethanol (1.5 mL), 10% aluminium chloride (0.1 mL) and 1 M potassium acetate (0.1 mL) were added to individual sample extracts (0.1 mL) before incubating in the dark for 40 min. The absorbance of the samples and standards was measured at 415 nm (UV-2600; Shimadzu, Kyoto, Japan). Standard curve for analysing TFC of the samples was prepared by using a stock solution of 0.2 mg/mL quercetin. The analysed samples were expressed on dry mass basis in mg quercetin equivalents (QE) per g sample.

### Total anthocyanin content

For determining total anthocyanin content (TAC), pH differential method was used with some modifications (*26*). Sorghum bran extract samples (1 mL) were diluted with the respective solvents and incubated for 2 h at 25 °C in dark. Luteolin and apigenin contents in the samples were measured at 270 nm. Finally, the anthocyanin content was estimated after determining the absorption coefficient of both luteolin and apigenin at 270 nm (UV-2600; Shimadzu) by rearranging the Beer-Lambert’s law (*27*):

*γ*=*A*/*ε*·*l*·10^3^·*M*·DF /1/

where *A* is the absorbance at 270 nm, *ε* is molar absorption coefficient (M^-1^ cm^-1^), *l* is light path length (1 cm), *M* is the molecular mass of anthocyanin standards, and DF is dilution factor.

Total anthocyanin content of sorghum bran extracts was expressed as the concentration (mg/mL) with respect to the concentration of apigenin and luteolin.

### Total condensed tannins

Total condensed tannins (TCT) in all the sample extracts were estimated *via* butanol-HCl method (*28*). The samples (0.5mL) were added to butanol-HCl (3 mL) and iron(III) reagent (0.1 mL iron(III) ammonium sulphate in 2 M HCl) solution before incubating at 100 °C for an hour. Then, absorbance was measured at 450 nm (UV-2600; Shimadzu). Standard curve for analysing TCT of the samples was prepared by using a stock solution of 5 mg/mL catechin. The TCT concentrations were expressed on dry mass basis in mg catechin equivalents (CE) per g sample.

### Antioxidant activity

The antioxidant activity of the sorghum bran extracts was measured by three different assays (*29*–*31*), namely: DPPH scavenging activity, ABTS radical cation decolourization assay and CUPRAC. The scavenging activity was calculated for DPPH and ABTS assays as:

Scavenging activity=[(*A*_c_-*A*_s_)/*A*_c_]·100 /2/

where *A*_c_ is the absorbance of control samples and *A*_s_ is the absorbance of extract samples.

The antioxidant activity of pure luteolin and apigenin solubilised in methanol individually was also investigated at a concentration of 1 mg/mL, with methanol being both the reference and control.

### DPPH scavenging activity

The sorghum bran extracts (100 μL) were reacted with 2.9 mL of 0.1 mM DPPH-methanol solution and were incubated for 30 min in the dark, before measuring the absorbance at 517nm (UV-2600; Shimadzu). Methanol was taken as reference, whereas control contained respective solvents used for extraction: water, anhydrous methanol, acidified methanol and 1% [BMIM]Cl instead of sample extract. Trolox (1 mg/mL) was used as the standard. Samples were analysed and expressed on dry mass basis in μmol Trolox equivalents (TE) per g sample.

### ABTS radical cation decolourization assay

For ABTS˙^+^ generation from ABTS salt, 3 mM K_2_S_2_O_8_ and 8 mM ABTS salt were reacted in distilled water for 16 h at room temperature in the dark. The ABTS˙^+^ solution was then diluted with absolute methanol to obtain an absorbance of 1.5 at 730 nm (UV-2600; Shimadzu). The sorghum bran extracts (100 μL) were reacted with 2.9 mL of fresh ABTS˙^+^ solution, incubated for 30 min in the dark and then the absorbance was measured against anhydrous methanol, acidified methanol or 1% [BMIM]Cl as references for respective solvent extracts. Trolox (1 mg/mL) was used as the standard. Samples were analysed and expressed on dry mass basis in μmol TE per g sample.

### Copper(II)ion reducing antioxidant capacity (CUPRAC) assay

The sample extracts (100 μL) were added to 10 mM copper chloride solution (1 mL), 7.5 mM neocuproine alcoholic solution (1 mL) and 1 M ammonium acetate buffer solution (pH=7). The final volume was made upto 4.1 mL before incubating for 30 min in the dark. Copper chloride and ammonium acetate buffer solution was taken as reference, whereas control contained respective solvents of extracts: anhydrous methanol, acidified methanol, water and 1% [BMIM]Cl instead of the sample extract. Trolox (1 mg/mL) was used as the standard. The samples and standards were analysed at 450 nm (UV-2600; Shimadzu). The corrected absorbance of the samples (*A*_sample_-*A*_blank_) were extrapolated in the Trolox standard solution graph and thereafter represented on dry mass basis as µmol TE per g sample.

### Antibacterial activity

#### Bacterial strains and growth conditions

Glycerol stock (30%) cultures of *Staphylococcus aureus* NCDC109 and *Escherichia coli* ATCC5922 were used in the present study. The inocula were prepared by incubating the cultures (both *E. coli* and *S. aureus*) in nutrient broth (NB) and tubes were incubated at 37 °C overnight with continuous shaking (1×*g*) (MaxQSHKE6000 incubated shaker; Thermo Fischer Scientific, Cincinnati, OH, USA). Then, the culture was streaked onto eosin methylene blue and nutrient agar plates respectively and the plates were incubated (Forma 4111TS incubator; Thermo Fischer Scientific) at 37 °C for 24 h. The strains were then subcultured under the same conditions for further analysis.

#### Well diffusion assay

Well diffusion assay was performed as described by Kil *et al.* (*32*). The dry extracts were re-suspended into their respective solvents to yield a final concentration of 12 mg/mL. Pure standards apigenin and luteolin dissolved in methanol were analysed at the same concentration as dry extracts (12 mg/mL). The volumes of 40 and 100 μL of all four extract solutions, as well as standards and solvent controls (water, methanol, acidified methanol and ionic liquid) were taken to test the antimicrobial activity. Fresh cultures of *E. coli* and *S. aureus,* with the absorbance of 0.1 at 600 nm, were flooded onto nutrient agar plates. Equidistant wells (2 mm in diameter) were created in each plate to test the two volumes of the prepared extracts. The plates were then incubated (Forma 4111TS incubator; Thermo Fischer Scientific) at 37 °C for 24 h to determine the inhibition zone. The inhibition zones were observed and the diameter was measured with a simple ruler. The assay was performed in triplicates. Since pure standards (apigenin and luteolin), water, methanolic and ionic liquid extracts did not show the presence of zones of inhibition, minimum inhibitory concentration (MIC) assay for these was not performed.

#### Minimum inhibitory concentration assay

This analysis was performed to determine the minimum inhibitory concentration (MIC) of the extract prepared from acidified methanol against *S. aureus* and *E. coli* as previously described (*33*, *34*). Briefly, logarithmically grown cultures of *E. coli* and *S. aureus* were utilised for this study. Both cultures were treated with sorghum bran extract solutions at various concentrations (72.5-0.25 mg/mL). Twofold serial dilution of the extract was performed in NB medium and bacterial culture inocula were added (10^6^ CFU/mL). After incubation (Forma 4111TS incubator; Thermo Fischer Scientific) at 37 °C for 24 h, serial dilutions (72.5, 36.2, 18.1, 9.0, 4.5, 2.2, 1.1, 0.5 and 0.25 mg/mL) were pipetted on nutrient agar plates to determine MIC by calculating the CFU/mL. The assay was performed in triplicates.

### Statistical analysis

Duncan’s multiple range tests *via* IBM SPSS Statistics software v. 20.0 (*22*) was used for evaluating significant differences among a set of mean values of TPC, TFC, TCT and TAC as well as for the different assays performed to estimate the antioxidant activity of all extracts. All antibacterial analysis data were presented as mean value±standard deviation wherever appropriate. All the graphs were plotted using the OriginPro 2019 v. 9.60 software (*35*).

## RESULTS AND DISCUSSIONS

### Extraction yield

Repeated extraction of sorghum bran (2.5 g) in the four solvents was performed. Dry yields of anhydrous methanol, acidified methanol and water solvent extracts were estimated to be (2.9±0.5), (11.7±1.4) and (4.9±0.1) % respectively, whereas for ionic liquid (IL) the yield was (14.9±0.7) %. Thus, it is observed that the yield from ionic liquid extract (ILE) was the highest, followed by acidified methanol extract (AME) and water extract, whereas the yield of anhydrous methanol extract (ANME) was the lowest among all. The colour of the extracts indicates that anhydrous methanol, water and IL extracts possibly consist of similar bioactive compounds, while acidified methanolic extract contains a fair quantity of anthocyanins among the bioactive components. The highest yield of sorghum bran using ionic liquid justified its employability as an extraction solvent. Various interactions like Van der Waal’s forces, dispersive, hydrogen bonding, dipole-dipole, ionic/charge-charge and π-π/n-π can occur between the ionic liquid and the solutes, and therefore it is considered a good solvent for extraction (*36*, *37*). Moreover, it shows significant differences in polarities that can be adjusted by changing the anion or cation, and hence is a versatile solvent for a wide variety of compounds such as polyphenolic molecules (*38*). Polar protic, aromatic compounds are highly soluble in ionic liquids, which helps the selective extraction of various molecules. Protic polyols form hydrogen bond with the electronegatively charged IL anion, whereas aromatic molecules having delocalised π electron clouds produce electrostatic field in the interaction with IL cation (*38*).

### Effect of pH

**Fig. S1:**
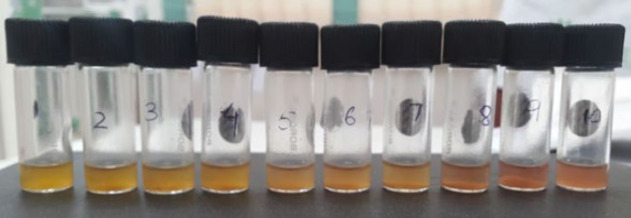
Influence of pH on the anhydrous methanolic extract of sorghum bran

There was no visible change in the colour with the variation of pH on water extract and ILE, which further suggests that anthocyanin concentration might be possibly quite low in these extracts. On the other hand, the increase of the pH of ANME caused significant colour change. From Fig. S1, a visible colour change from orange-yellow to pinkish-red is observable as the pH of the ANME increases (pH>4), thereby suggesting possible structural modifications of the bioactive components of the bran extract. The colour change of methanolic extract from acidic to alkaline pH (orange-yellow to pinkish-red) suggests the presence of anthocyanins that gradually transform and modify as the pH approaches alkaline environment (*5*, *39*). Thus, the properties of the ILE and water extract are similar and differ significantly from the ANME as evident with pH change.

Although AME contains a substantial amount of anthocyanins, as evident from the colour of the extract as well as earlier reports (*20*), no colour change with pH variation wasobserved. This is because the extracted anthocyanins do not react with the solvent (acidified methanol), whereas the same is not true in the case of anhydrous methanol, similar to aqueous acetone, where solvent-anthocyanin reaction occurs unless the extraction parameters (time and temperature) are strictly maintained (*5*). Such pH modifications imparting a colour change in the natural extracts may be quite promising as sensors or indicators in the area of food quality and safety.

### Qualitative analysis by LC/MS/MS

**Fig. 1:**
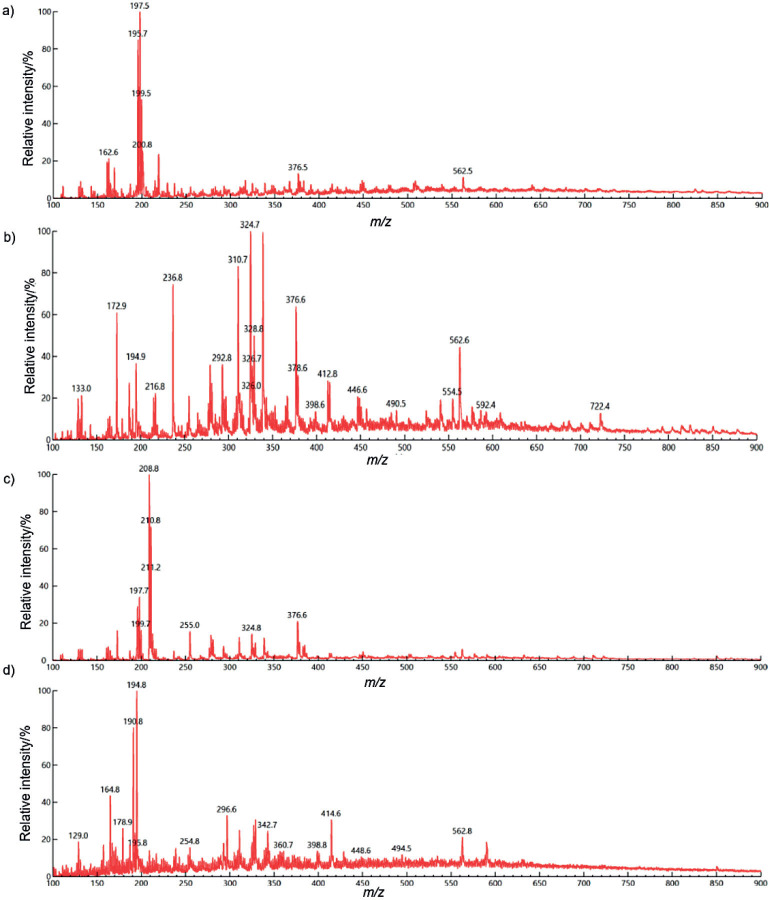
Liquid chromatography with tandem mass spectrometry analysis of sorghum bran extracts: a) acidified methanol, b) methanol, c) ionic liquid, and d) distilled water

Untargeted phenolic profiling of the sorghum bran extracts was performed (Fig. 1) and tentatively identified by comparing with the available mass spectra literature. Methanol and distilled water extracts were observed to have numerous peaks, suggesting greater number of compounds than ionic liquid and acidified methanolic extracts. Moreover, various mass peaks were found in more than one extract, indicating that similar compounds are present in the extracts. However, from the previously available data, we could identify a few compounds in all the extracts. Caffeic acid (*m/z*=178.9) (*22*–*24*, *40*), coumaroyl-caffeoylglycerol (*m/z*=398.8) (*41*) and coumaroyl-feruloylglycerol (*m/z*=414.6) (*41*) were found to be unique only to distilled water extract. Similarly, diferulic acid (*m/z*=324.8) (*21*) and coumaric acid (*m/z*=162.6) (*21*, *22*, *24*, *42*) were observed only in ionic liquid and acidified methanol extracts, respectively. Compounds such as *trans*-ferulic acid, isoferulic acid (*m/z*=194.9) (*22*, *24*), tricin-O-hexoside (*m/z*=490.5) (*41*) and unidentified procyanidin glycoside (*m/z*=722.4) (*27*) were identified in methanol extract. Various derivatives of apigenin and luteolin have also been observed in all the extracts, such as apigeninidin in distilled water (*m/z*=254.8) and ionic liquid (*m/z*=255) extracts (*22*, *23*, *41*, *43*), luteolin-7-O-glucoside in distilled water (*m/z*=448.6) (*42*) and methanol (*m/z*=446.6) (*27*, *41*) extracts and 6-C-pentosyl-8-C-hexosyl apigenin in distilled water (*m/z*=562.8) (*27*, *41*), methanol (*m/z*=562.6) (2*7,41*) and acidified methanol (*m/z*-562.5) (*41*) extracts.

### Determination of TPC, TFC, TCT and TAC

Polyphenols are the most important group of phytochemicals not restricted only to their significant role in the appearance (colour) of the plant, but for their nutraceutical characteristics such as antioxidant and antimicrobial activity, anti-inflammatory and anticancerous properties, which are not only beneficial to human health but also act as a defence mechanism of the plant producing them. Therefore, their quantification in entirety and individually is important to obtain a fair idea about the polyphenolic composition imparting such varied functional properties.

In our study of sorghum bran extracts with different solvents, TPC as GAE was found to be highest in AME (26.8 mg/g) followed by water extract (15.2 mg/g), while in the anhydrous methanolic (ANME) and ionic liquid (ILE) extracts, TPC was found to be comparable at the values of 9.0 and 7.4 mg/g, respectively (Table 1), which is also corroborated *via* the statistical analysis, indicating no significant difference between the TPC of the two extracts. A similar phenolic concentration (*20*) as well as the trend were observed when acidified methanol extract had higher phenolic and flavonoid content (*8*, *25*). Lower total phenolic concentration of sorghum bran extracted in aqueous methanol than of anhydrous methanol extract was obtained in the present study (*44*).

**Table 1:** Total phenol content (TPC), total flavonoid content (TFC) and total condensed tannin (TCT) content in sorghum bran extracts

Sample	*w*(TPC as GAE)/(mg/g)	*w*(TFC as QE)/(mg/g)	*w*(TCT as CE)/(mg/g)
WE	(15.2±0.2)^a^	(7.4±0.4)^a^	(83.1±2.9)^a^
ANME	(9.0±0.8)^b^	(6.9±0.3)^a^	(35.3±1.2)^b^
AME	(26.78±1.0)^c^	(14.2±0.4)^b^	(58.6±2.4)^c^
ILE	(7.4±0.7)^b^	(3.0±0.1)^c^	(63.2±2.1)^d^

Mean value±S.D. (*N*=3). Letters in the same column indicate the significant differences (p<0.05).

GAE=gallic acid equivalent, QE=quercetin equivalent, CE=catechin equivalent, WE=water extract, ANME=anhydrous methanol extract, AME=acidified methanol extract, ILE=ionic liquid extract

Contrary to the TPC, TFC as QE in ILE was quite low (3.0 mg/g) compared to ANME and water extract, where it was similar at 7.4 and 6.9 mg/g, respectively. AME had the highest flavonoid content (14.2 mg/g), possibly due to high concentration of anthocyanins, which will be disclosed later.

Condensed tannins as CE were found to be the highest in the water extract (83.1 mg/g) followed by ILE (63.2 mg/g), and AME (58.6 mg/g), while ANME had the lowest concentration of condensed tannins at 35.3 mg/g. Condensed tannins in ILE and AME may appear to be in the similar range, but statistical analysis showed significant differences among all the extracts.

The higher extraction of tannins in ionic liquid could be due to the high solvency of phenols in ionic liquid and/or their deprotonation in the dissolution reaction by ionic liquid (*45*). Another possibility is the modification of condensed tannins, which led to the synthesis of condensed tannin-ester derivatives carried out by hydroxides or organic bases (amines, ionic liquids or pyridines) (*46*). Hydrophobicity of BMIM[Cl] has the ability to increase the extraction efficiency of targeted secondary metabolites (*37*, *47*).

**Fig. S2:**
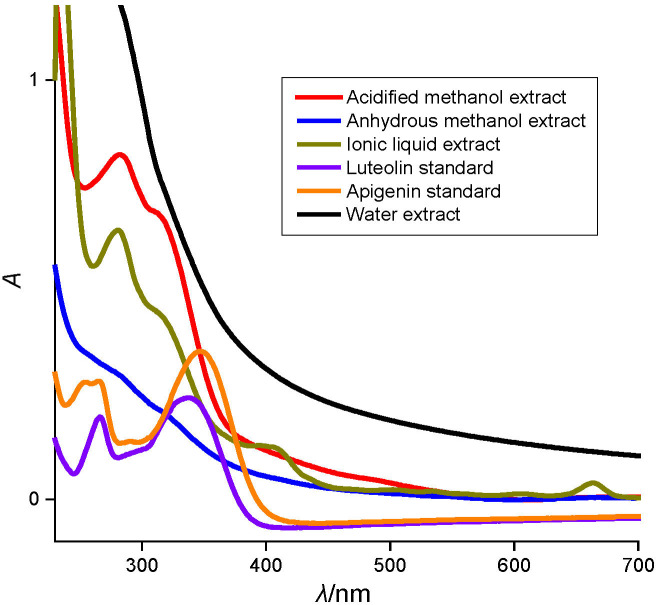
Absorption spectra of the sorghum bran extracts and standards (apigenin and luteolin, *γ*=0.1 mg/mL)

The absorbance spectra of all the extracts show substantial absorption in the visible region with maxima at 270 and 345 nm (Fig. S2) indicating the presence of apigenin and luteolin, which are two of the major anthocyanins known to be present in the sorghum bran (*5*).

The total anthocyanin content (TAC),determined in all the four extracts with respect to apigenin and luteolin at 270 nm (*27*) (with molar absorption coefficients (*ε*) 7540.5 and 5764.7 M^-1^ cm^-1^, respectively), was the highest in AME, followed by ANME (Table 2). A similar result was reported for the brown sorghum bran samples extracted in acidified methanol (*5*).The anthocyanin concentration of water extract and ILE was found to be quite similar (apigenin and luteolin in water extract: (178.0±7.2) and (246.9±10.0) mg/L and in ILE: (168.9±7.2) and (234.1±10.0) mg/L, respectively), which was confirmed by statistical analysis as well. The low concentration of anthocyanins in the ILE compared to ANME possibly contributes to the low flavonoid concentration, but the presence of higher condensed tannins explains the comparable phenolic concentration of ILE with ANME.

**Table 2:** Total anthocyanin concentration (TAC) of sorghum bran extracts expressed as concentration of apigenin and luteolin

Sample	*γ*(TAC as apigenin)/(mg/L)	*γ*(TAC as luteolin)/(mg/L)
AME	(269.7±9.1)^a^	(373.9±12.5)^a^
ANME	(235.6±5.1)^b^	(326.6±7.1)^b^
WE	(178.0±7.2)^c^	(246.9±10.0)^c^
ILE	(168.9±7.2)^c^	(234.1±10.0)^c^

Mean value±S.D. (*N*=3). Letters in the same column indicate the significant differences (p<0.05). AME=acidified methanol extract, ANME=anhydrous methanol extract, WE=water extract, ILE=ionic liquid extract

### Determination of antioxidant activity

Assays based on different mechanisms that are relevant to the complex matrix are able to better portray the *in vitro* system and thereby *in vivo* systems (*19*), whereas there may not be close correspondence among the results due to various factors being entirely different such as the reaction mechanism/kinetics, solvent dependency, oxidation potential, *etc* (*48*). Keeping the above in mind, the antioxidant activity of all the four sorghum bran extracts using DPPH, ABTS and CUPRAC assays was investigated. Table 3 shows that the antioxidant activity of pure apigenin and luteolin is quite low when compared to the extracts. However, between these two, luteolin was observed to have significantly higher antioxidant activity than apigenin, which is due to the presence of 3,4-dihydroxyl groups in the B-ring, making it appropriate for a faster oxidation than apigenin, having only a single hydroxyl group in its B-ring. The presence of 3,4-dihydroxyl groups in the B-ring in luteolin leads to enhanced stability, thereby increasing the scavenging activity towards free radicals (*49*). ILE, measured as Trolox equivalents on dry mass basis, was comparable (63.2–100.8 μmol/g) with water extracts (62.9-115.0 μmol/g), while it was low compared to the anhydrous methanolic extract, although statistically all the extracts were observed to be significantly different from each other. The trend of the scavenging activity of these extracts *via* DPPH and ABTS assays exhibited comparable results.

**Table 3:** Antioxidant activity of sorghum bran extracts, apigenin and luteolin measured with DPPH, ABTS and CUPRAC assays

Sample	DPPH	(*n*(Trolox)/*m*(sample))/(μmol/g) ABTS	CUPRAC
Water extract	(62.9±0.7)^a^	(115.0±0.8)^a^	(87.6±0.7)^a^
ANME	(23.1±0.7)^b^	(75.6±1.3)^b^	(24.6±1.3)^b^
AME	NA	(88.8±2.5)^c^	(106.8±1.8)^c^
ILE	(85.2±1.2)^c^	(100.8±0.9)^d^	(63.2±1.9)^d^
Apigenin	(0.004±0.006)^d^	(0.2±0.02)^e^	(0.1±0.1)^e^
Luteolin	(6.3±0.1)^e^	(7.7±0.8)^f^	(6.1±0.5)^f^

Mean value±S.D. (*N*=3). Letters in the same column indicate significant differences (p<0.05).

NA=not applicable, WE=water extract, ANME=anhydrous methanol extract, AME=acidified methanol extract, ILE=ionic liquid extract

Qualitative analysis of extracts *via* LC/MS/MS shows a variety of polyphenols, as is observable in other reported literature (*21*–*24*). The presence of such a wide range of polyphenols, both qualitatively and quantitatively, as well as the variation in principle of the performed assays attributes to the diverse antioxidant activity. The results indicate that although TFC is the lowest in ILE, its antioxidant activity is comparable to the water extract and quite higher than the ANME. This is because the total phenol content of ILE is substantial enough and it is the fair amount of condensed tannins that justifies reasonable antioxidant activity (*50*). In the case of ANME, although TPC is comparable with ILE and TFC is higher in ANME than in ILE, relatively low concentration of condensed tannins in ANME possibly justifies its lowest antioxidant activity. Thus, the presence of high concentration of condensed tannins both in the ionic liquid extract and water extract contributed to the strong antioxidant activity compared to conventional organic solvents like anhydrous methanol or acidified methanol extracts.

**Fig. 2:**
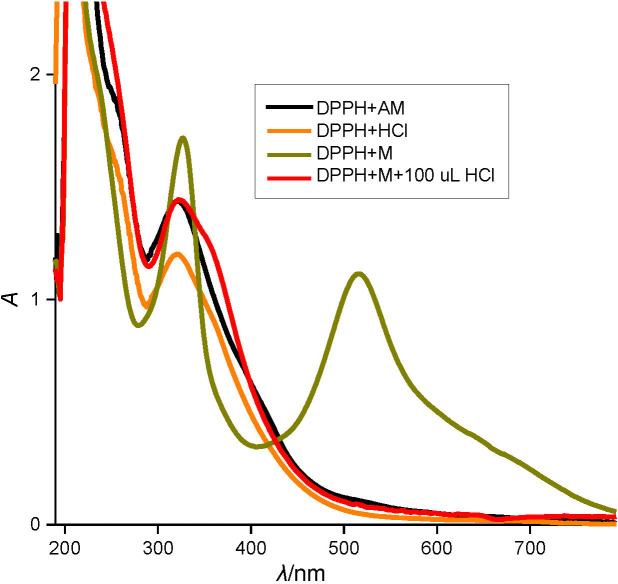
Influence of HCl (*φ*=0.01) on the absorption spectra of DPPH. AM=acidified methanol, M=methanol

There are reports that require special mention here where antioxidant activity of acidified extracts of sorghum was determined by DPPH assay (*5*, *25*), and also a few other studies (*50*, *51*) where it was done either by changing the solvent or the pH of the solvent. In this work, the nature of DPPH band at 517 nm has been studied in the presence of an acid. Fig. 2 clearly indicates that there is complete quenching of the absorption band with the addition of 1% HCl. Similar was the case with 1% acidified methanol. Therefore, it can be ascertained that evaluation of antioxidant activity by DPPH assay of acidified methanolic extract is not possible in contrast to earlier reports on the same.

### Determination of antibacterial activity

#### Antibacterial activity by well diffusion assay

**Fig. 3:**
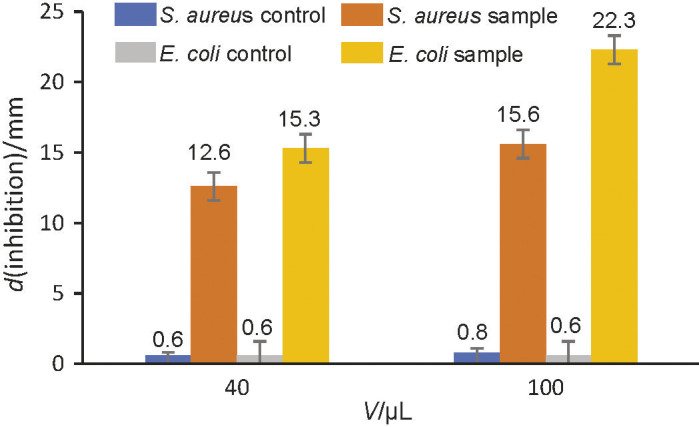
Zone of inhibition of acidified methanolic sorghum bran extract against *S. aureus* and *E. coli*

This assay was performed for all the four extracts against both test organisms, *Escherichia coli* and *Staphylococcus aureus*. The concentration of all extracts (acidified methanolic, anhydrous methanolic, ionic liquid and water extracts) as well as the pure standards (apigenin and luteolin), added in each well, was kept uniform to 12 mg/mL. The only extract where the inhibition zone was clearly visible was the acidified methanolic extract against both *Escherichia coli* and *Staphylococcus aureus*; the inhibition zone was absent in the case of the other three extracts, namely ionic liquid, water and anhydrous methanolic extract. The average zone of inhibition against *S. aureus* was 12.6 and 15.6 mm in the acidified methanolic extract in volumes of 40 and 100 μL, respectively. Inhibition zone of acidified methanolic extract in volumes of 40 and 100 μL, against *E. coli* were observed to be 15.3 and 22.3 mm, respectively. The well diffusion assay results indicate that sorghum bran acidified methanolic extract is more effective in controlling *E. coli* than *S. aureus.* The control plate containing only solvent (acidified methanol) also exhibited zone of inhibition (0.6 and 0.8 mm with 40 and 100 μL of the extract, respectively, of *S. aureus,* and 0.6 mm with both volumes in the case of *E. coli*), which was, however, quite small compared to the acidified methanolic extract (Fig. 3).

Similar results have been reported (*16*) for methanolic extracts of sorghum grains, where 9 out of 10 sorghum varieties showed maximum zone of inhibition against *E. coli* (14-30 mm). On the other hand, significant antibacterial effect of defatted sorghum flour extract was observed (*17*) against *S. typhimurium, S. aureus* and *B. subtilis* by disk diffusion method at a lower concentration (6.25 mg/mL), whereas inhibition zones against *E. coli* were found at a higher concentration of the extract (25 mg/mL). Since there was the absence of inhibition zones for methanolic, water and IL extracts, it may be concluded that there is no antibacterial activity at this concentration (12 mg/mL) of the extracts against *S. aureus* and *E. coli.*

In our work, the pure standards (apigenin and luteolin) did not exhibit any antibacterial activity against the tested microorganisms at a concentration of 12 mg/mL, which has also been reported previously for pure apigenin (200 μg/mL) against *S. aureus* (*52*, *53*). Luteolin and its derivatives, derived from plant extracts, have been reported to have antibacterial and antiviral activity, whereas reports of such activity in pure luteolin are not specified (*54*, *55*). Mostly, these compounds or their derivatives have been isolated from plant origins that have been observed to have antimicrobial activity, indicating the role of other bioactive compounds as well as a symbiotic relationship between the compounds enhancing each other’s functional activities (*53*, *55*, *56*). Dedicated work on pure compounds found in plant extracts (however, not isolated from plants) imparting various functional properties, especially antimicrobial activity, is quite limited (*57*) and hence, should be studied to rightly attribute the properties to the investigated compounds.

Recently, there has been a surge in the investigation of functional properties of plant extracts rich in bioactive compounds. However, the in-depth scrutiny of the same has not been achieved due to the diversity in plant compounds, solvents, extraction as well as functional activity (antimicrobial *etc.*) procedures. This varied degree of probe has led to the stage of partial knowledge, which is useful but also insufficient at the same time.

Keeping in mind the variation in the parameters that play a critical role in imparting antimicrobial properties such as various assays used along with differing solvents, extraction procedures, various plant species, their extracts and concentrations, purity and origin of bioactive compounds, it is difficult and logically not ideal to compare with previously reported data. Furthermore, with respect to antimicrobial activity, the type of microbial strain also plays a vital part. According to literature, some strains are more sensitive to plant extracts and compounds than others (*57*). Here, the interaction between the bioactive compounds and the microbial strain plays a major role that causes great discrepancies in the results from one investigation to another.

#### Determination of minimum inhibitory concentration

**Fig. 4:**
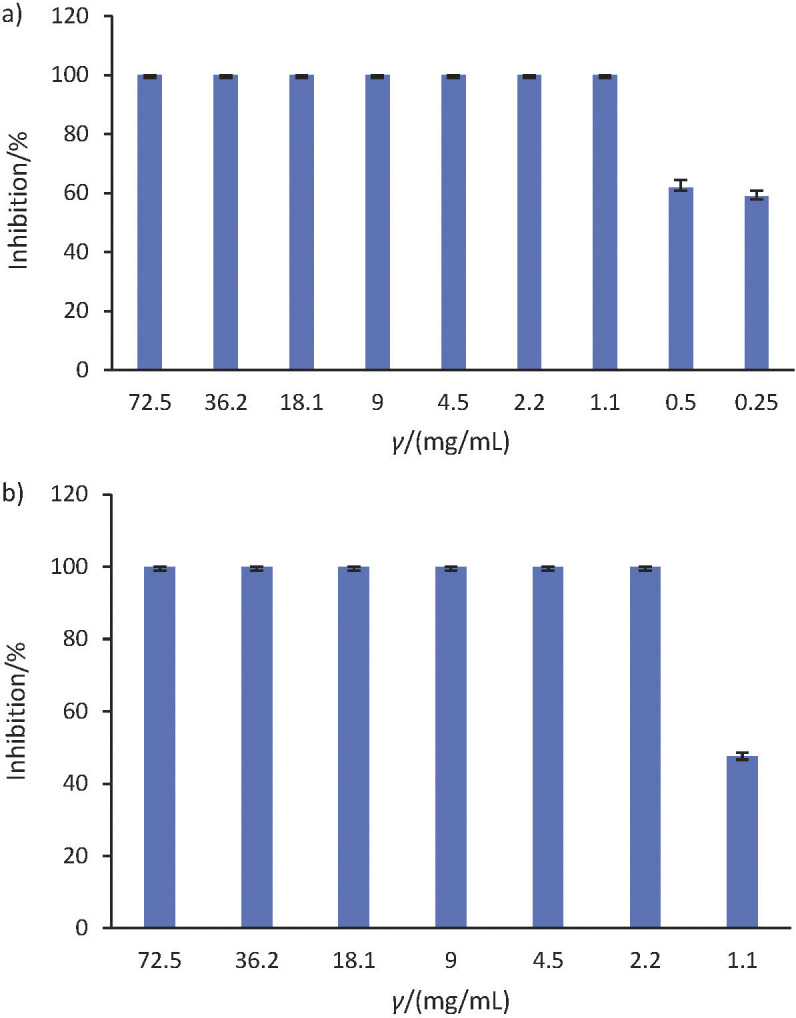
Minimum inhibitory concentration (MIC) assay for acidified methanolic extract against *S. aureus* and *E. coli* respectively

MIC assay was performed with acidified methanolic extract sample to find the lowest concentration where killing effect would be observed against the test organisms. Total inhibition of *S. aureus* was observed at the minimum concentration of 1.1 mg/mL (Fig. 4a), whereas the 100% inhibition of *E. coli* was observed at a minimum concentration of 2.2 mg/mL (Fig. 4b). As per the above observations of MIC results, lower concentration of acidified methanolic extract of sorghum bran was found to be more effective against Gram-positive bacteria (*S. aureus*) than Gram-negative bacteria (*E. coli*).

In another study, a similar observation was reported where saponin extracts of sorghum grains were found to be effective in inhibiting Gram-positive bacteria (*S. aureus*) at concentrations of 50 and 25 mg/mL, but ineffective against fungi and *E. coli* (*15*). A comparable MIC trend of sorghum distillery residues was observed (*18*), where the extracts (alcohol, cold water and hot water) were quite effective against *Bacillus cereus*, *Escherichia coli* O157:H7 and *Salmonella* spp. but less effective against *Staphylococcus aureus*. The minimum inhibitory concentration of alcohol and water extracts was found to be between 4-6 mg/mL, which is much higher than the MIC of acidified methanol extract against both *S. aureus* and *E. coli* in the present work.

Antimicrobial properties of 25 cultivars of sorghum (extracted in methanol and fractioned in *n*-hexane, water, *n*-butanol and ethyl acetate) were investigated and it was observed that extracts of two cultivars (Bulkeunchalsusu and Neulsusu) had better MICs (0.50 and 0.25 mg/mL) than other cultivars against almost all tested microorganisms (*K. pneumonia, S. aureus, C. albicans, Salmonella typhimurium, Bacillus subtilis* and *E. coli*). Overall, strongest inhibitory effect of sorghum cultivars and their fractions was seen against *E. coli* and methanol extract was proven to be most effective against all microbes (*32*). This result was contrary to our observations as no inhibition zone was observed when using methanol sorghum bran extract.

## CONCLUSION AND FUTURE SCOPE

This work reveals that the extraction of bioactive compounds primarily depends on the nature of the used solvent. Unlike acidified methanolic extract, the effect of anhydrous methanol extract on colour change was due to the extracted anthocyanin susceptibility to solvent modification, and therefore found to be sensitive to the pH change. Such pH modifications imparting a colour change in the natural extracts may be quite promising for their use as sensors or pH indicators.

Qualitative analysis of the extracts revealed the presence of several phenolic compounds consisting of phenolic acids, flavones and 3-deoxyanthocyanidins, which have been previously proven to impart functional properties such as antioxidant and antimicrobial activity synergistically. This synergism was also observed in the present work, ascertaining that individual purified compounds were less functional than the crude extracts from the different solvents.

The most significant aspect brought into light by this work is the role of ionic liquid in the extraction of sorghum bran antioxidants. It is quite evident from this study that sufficient quantity of bioactive compounds with strong antioxidant activity may be isolated utilizing ionic liquid in comparison to conventional extraction solvents. On the other hand, there is a dire requirement of an exhaustive study of the effect of ionic liquids with different cationic chain length, and anions as they strongly influence the water miscibility of ionic liquids, whereas the alkyl chain length of the cation affects the extraction yield efficiency. Furthermore, different extraction methods, like microwave-assisted or ultrasound-assisted extraction also need to be explored in combination with different ionic liquids for higher yields and functional activity of sorghum bran.

It is to be noted that a thorough and comparative assessment of antioxidant activity with the previous studies was not possible and logically reasonable due to various factors, like difference of sorghum bran species (differing demographic and environmental influences), nature of extraction solvents and revised extraction procedures as well as variation in the antioxidant and antimicrobial assays performed with modified protocols.

The variation in the minimum inhibitory concentrations in the antibacterial analysis may be responsible for the difference in the phenol composition of the bran and sorghum grain as well as for the presence of secondary metabolites that play a vital role in plant defence against pests and pathogens. Moreover, solvents used for extraction of compounds also play a significant role, as the nature of the solvent aids in the extraction of specific compounds from the entire matrix. Another reason may be the difference in the sensitivity of Gram-negative and Gram-positive microbes, due to their structural dissimilarities, against the bioactive compounds and their sources. Further extensive research into the identification and isolation of bioactive compounds from such agricultural waste and by-products can lead to interesting innovations in the near future.
